# miR-146b-5p Suppresses Btg2 to Promote Fibroblast Proliferation and Is Associated with Prrx2 and Myh10

**DOI:** 10.3390/biom16101407

**Published:** 2026-09-28

**Authors:** Chuncheng Liu, Wenhua Pu, Rong Wang, Yingxin Liu, Xinyang Li, Lu Cai, Hongyu Zhao

**Affiliations:** Inner Mongolia Key Laboratory of Life Health and Bioinformatics, School of Life Science and Technology, Inner Mongolia University of Science & Technology, Baotou 014020, China; liuchuncheng@imust.edu.cn (C.L.);

**Keywords:** silicosis, miR-146b-5p, Btg2, fibroblast proliferation

## Abstract

Pulmonary fibrosis is a chronic progressive lung disease characterized by abnormal proliferation and activation of fibroblasts. However, the regulatory mechanisms of miRNAs and their target genes on fibroblast proliferation during silicosis (silica-induced pulmonary fibrosis) remain incompletely understood. A mouse silicosis model was established by dynamic dust inhalation, and lung tissues were collected at 7, 14, 28, and 56 days after dust exposure, followed by multi-time-point miRNA sequencing and transcriptome sequencing. Using integrated omics, bioinformatics analysis, and molecular interaction validation, the key miRNAs and their target genes regulating fibroblast proliferation were screened and verified. The results showed that time-series clustering and H&E staining confirmed successful modeling of the transition from acute inflammation to chronic fibrosis induced by SiO_2_. Differentially expressed miR-146b-5p during SiO_2_ stress may affect the cell cycle of fibroblasts by regulating Btg2. Mechanistically, IP-MS combined with Co-IP identified interactions between Btg2 and Prrx2 or Myh10, suggesting their potential involvement in this regulatory process. These findings provide new experimental evidence for the molecular mechanisms of silica-induced pulmonary fibrosis in a mouse model, and may offer insights for understanding the pathogenesis of human silicosis.

## 1. Introduction

Pulmonary fibrosis (PF) is a chronic respiratory disease characterized by progressive fibrosis of the lung parenchyma [1]. Its fundamental pathological process involves repeated injury to the lung epithelium, accompanied by fibroblast accumulation, myofibroblast activation, and extracellular matrix deposition [2]. Among various etiologies, silicosis, an irreversible occupational lung disease caused by inhalation of silica dust, poses a major global health burden. It is estimated that approximately 230 million workers worldwide are exposed to silica dust, and silicosis accounts for about 90% of all pneumoconiosis cases [3]. Compared with other fibrosis models, such as bleomycin-induced fibrosis, silica-induced pulmonary fibrosis has a distinct pathogenesis. In this process, silica dust particles promote aberrant proliferation and activation of fibroblasts mainly through persistent tissue irritation and inflammatory responses, rather than through direct cytotoxicity-induced tissue injury [4]. Fibroblasts and myofibroblasts play a central role in this process [5]. Previous studies have shown that during fibrosis, fibroblasts and myofibroblasts exhibit aberrant epigenetic regulation [6,7], resistance to apoptosis [8], and enhanced invasiveness [9].

Fibroblast activation, proliferation, and apoptosis resistance are critical events during fibrogenesis. Targeting these processes, especially the underlying molecular mechanisms, is therefore of great importance. Btg2, an important member of the BTG/Tob family, participates in multiple levels of regulation including transcription, post-transcriptional modification, and translation, and plays a significant regulatory role in cell proliferation and apoptosis [10]. For example, Btg2 is a critical factor in maintaining T cell quiescence, and Btg1 and Btg2 double-deficient T cells exhibit enhanced proliferation due to increased mRNA abundance [11]. Btg2 can also bind to histone deacetylases (such as HDAC1, HDAC4, and HDAC9) to inhibit Ccnd1 expression, thereby inducing G1 phase cell cycle arrest [12]. However, the regulatory role of Btg2 in fibroblast proliferation requires further investigation.

The canonical TGF-β1/Smad signaling pathway plays a critical role in fibrosis by influencing myofibroblast differentiation, epithelial–mesenchymal transition, and endothelial-mesenchymal transition [13]. Upon activation of TGF-β type I receptor kinase by TGF-β1 signaling, Smad2 and Smad3 are phosphorylated. The activated Smad2 and Smad3 form a complex that associates with the common mediator Smad4 and translocates into the nucleus, thereby regulating the expression of target genes [14]. Similar to TGF-β, miRNAs also play important regulatory roles in the process of fibrosis and in fibroblasts. Among them, the miR-29 family has received the most attention [15]. Besides miR-29, the miR-146 family (including miR-146a-5p and miR-146b-5p) also plays important roles in inflammatory and fibrotic diseases. miR-146a-5p and miR-146b-5p are encoded by genes on different chromosomes [16]. Although they differ in their induction mechanisms, both can participate in negative feedback regulation of NF-κB signaling by targeting IRAK1 and TRAF6 [17,18]. In fibrosis research, miR-146a-5p has been shown to exert antifibrotic effects by regulating TGF-β/Smad and other signaling pathways [19]. However, the role of miR-146b-5p in pulmonary fibrosis remains controversial. Some studies have reported that it is significantly upregulated in the peripheral blood of patients with silicosis [20], whereas other studies have shown that its expression is downregulated in lesional fibroblasts from patients with IPF and systemic sclerosis [21], suggesting that its role may be cell type- or disease stage-specific.

Numerous studies have confirmed the important role of miRNAs in pulmonary fibrosis. However, most focus on a single time point or a specific stage, and the dynamic changes in miRNA expression over time remain poorly understood. Compared with single-time-point differential expression analysis, multi-time-point analysis can characterize the dynamic changes in gene expression during disease progression, thereby identifying key molecules that exert sustained or stage-specific regulatory roles in the development and progression of fibrosis. In this study, a mouse model of pulmonary fibrosis was established using a dynamic dust exposure system. Through multi-time-point miRNA sequencing and transcriptome sequencing, we systematically screened differentially expressed miRNAs and their potential target genes during disease progression, and identified *Btg2* as a key target. Furthermore, we demonstrated in fibroblasts that miR-146b-5p affects cell proliferation by regulating Btg2. These findings provide new experimental evidence and theoretical support for elucidating the molecular mechanisms of silica-induced pulmonary fibrosis in a mouse model, and may offer insights for understanding the pathogenesis of human silicosis.

## 2. Materials and Methods

### 2.1. Dynamic Dust Exposure in Mice

Animal housing: Male C57BL/6J mice were selected for this study based on the epidemiological characteristics of silicosis, which exhibits a significantly higher incidence and disease burden in males than in females [22]. Male specific-pathogen-free C57BL/6J mice, aged 8 weeks and weighing 20–24 g, were purchased from Spfbiotech (Beijing, China). Upon arrival, mice were allowed to acclimate to the housing conditions for 7 days before the start of the experiment. The mice were housed in a barrier facility under controlled conditions (temperature: 22–26 °C; relative humidity: 50% ± 5%; 12 h light/dark cycle). All mice had ad libitum access to food and water.

Dynamic SiO_2_ dust exposure: All mice were purchased from the same batch and housed under identical conditions. Mice were randomly assigned to the control and exposure groups. Mice assigned to the exposure group were placed in a dust exposure chamber (Shanghai TOW Intelligent Technology Co., Ltd., Shanghai, China) from 11:00 to 15:00 daily and exposed to SiO_2_ dust particles (S5631-500G, Sigma-Aldrich, St. Louis, MO, USA). The silica particles had a particle size distribution of 0.5–10 μm, with approximately 80% of particles between 1 and 5 μm, and a purity of ~99%. The dust was generated by a tablet-grinding dust generator with a fixed rotation speed of 0.20 rpm, and the air flow rate through the chamber was maintained at 7 L/min to ensure stable and consistent dust exposure across all experimental runs. Control mice were not exposed to dust and were housed under normal conditions. Mice subjected to SiO_2_ dust exposure were treated for 7, 14, 28, or 56 days. To minimize batch effects and ensure that all mice were sacrificed at a similar age and time point, the 56-day exposure group was started first, followed sequentially by the 28-, 14-, and 7-day groups, such that all exposure groups completed their respective exposure periods and were sacrificed within a similar time frame.

Inclusion and exclusion criteria: Mice meeting the age (8 weeks) and body weight (20–24 g) criteria were included in the study. No animals were excluded from the analysis during or after the exposure protocol.

Tissue collection: After dust exposure, mice were euthanized by cervical dislocation. Lung tissues were dissected and rinsed with physiological saline. The left lung was preserved in 4% paraformaldehyde, while the right lung was placed in liquid nitrogen.

### 2.2. Hematoxylin and Eosin (H&E) Staining

The left lung of each mouse was fixed in 4% paraformaldehyde fixative solution for at least 24 h, then dehydrated through an ethanol series, cleared with xylene, and embedded in paraffin before sectioning. After deparaffinization, the sections were stained with hematoxylin and eosin, followed by mounting. All sections were evaluated in a blinded manner with respect to group allocation.

### 2.3. Reverse Transcription Quantitative Real-Time PCR (RT-qPCR)

Total RNA extraction: A 20 mg mouse lung tissue sample was mixed with 200 μL of RNAiso Plus and homogenized on ice. After homogenization, an additional 800 μL of RNAiso Plus was added, and the mixture was mixed by inverting several times, followed by complete lysis on ice. For NIH3T3 cells, direct lysis was performed on ice after adding 1000 μL of RNAiso Plus. Then, chloroform was added to separate the organic and aqueous phases. Isopropanol was used to precipitate and isolate the RNA. The RNA quality was assessed using a UV spectrophotometer and agarose gel electrophoresis.

mRNA reverse transcription: Genomic DNA was first removed from the RNA samples using gDNA Eraser. Subsequently, 2 μg of total RNA was reverse transcribed into cDNA using PrimeScript RT Enzyme Mix I (RR047A, TaKaRa, Shiga, Japan). For miRNA reverse transcription, M-MLV reverse transcriptase (M1701, Promega, Madison, WI, USA) was used based on a stem-loop structure. The stem-loop primers for miRNA reverse transcription were designed using the sRNAPrimerDB software (User manual version 1.6).

miR-146b-5p (reverse transcription):

GTCGTATCCAGTGCAGGGTCCGAGGTATTCGCACTGGATACGACAGCCTAT.

U6 (reverse transcription): AACGCTTCACGAATTTGCGT.

Quantitative real-time PCR: The relative expression levels of key genes were detected using RT-qPCR. The relative expression of each gene was calculated using the 2^−ΔΔCt^ method. *Gapdh* was used as the internal control gene for normalization.

Btg2-F: GCGAGCAGAGACTCAAGGTT

Btg2-R: GGATCAACCCACAGGGTCAG

miR-146b-5p-F: CAGTGCAGGGTCCGAGGT

miR-146b-5p-R: ATGCGCGCTGAGAACTGAATT

U6-F: CTCGCTTCGGCAGCACA

U6-R: AACGCTTCACGAATTTGCGT

### 2.4. Western Blot

The basic procedure for Western blot was performed as described in a previously published study [23]. The protein extraction reagent was RIPA lysis buffer containing PMSF (RIPA:PMSF = 100:1). Mouse lung tissues were homogenized after adding the lysis buffer, followed by complete lysis on ice. For NIH3T3 cells, direct lysis was performed on ice after adding the lysis buffer. Insoluble components were removed by centrifugation. Protein samples were mixed with loading buffer and denatured by heating at 99 °C for 10 min. Proteins were separated by SDS-polyacrylamide gel electrophoresis (SDS-PAGE). After separation, proteins were transferred onto PVDF membranes, followed by blocking with 5% skim milk and overnight incubation with primary antibodies at 4 °C. The primary antibodies used were anti-Gapdh antibody (1:1000) (AF0006, Beyotime, Beijing, China), anti-Btg2 antibody (1:1000) (22339-1-AP, Proteintech, Wuhan, China), anti-Prrx2 antibody (1:1000) (ER60965, HUABIO, Hangzhou, China), and anti-Myh10 antibody (1:1000) (DF13164, Affinity Biosciences, Liyang, China). All antibodies used in this study were commercial products, and their suitability for Western blot has been validated by the suppliers. After incubation with HRP-conjugated goat anti-rabbit IgG (1:15,000) (ZB-2301, ZSBio, Beijing, China) or HRP-conjugated goat anti-mouse IgG (1:15,000) (ZB-2305, ZSBio, Beijing, China), protein expression levels were detected using the enhanced chemiluminescence (ECL) reagent (Beyotime, Beijing, China).

### 2.5. Cell Culture and Transfection

NIH3T3 and HEK293T cells were cultured in DMEM complete medium (DMEM: serum: penicillin–streptomycin = 900:100:10) and maintained at 37 °C in a humidified incubator with 5% CO_2_. Transfection was carried out using Lipo6000™ (Beyotime, Beijing, China). For transfection in a 6-well plate, 5 μL of Lipo6000™ transfection reagent and 250 μL of Opti-MEM reduced serum medium (Thermo Scientific, Waltham, MA, USA) were mixed in a 1.5 mL tube and incubated at room temperature for 5 min. In another 1.5 mL tube, 5 μL of miRNA mimics or gene-specific siRNA, or 2.5 μg of plasmid DNA, were diluted in 250 μL of Opti-MEM. The two solutions were combined, transferred to a 1.5 mL tube, and incubated at room temperature for 20 min. The entire mixture (510 μL) was then added to the culture well. After 5 h of transfection, the medium was replaced with fresh complete DMEM, and the cells were further cultured.

### 2.6. Vector Construction

Construction of gene overexpression vectors: The coding sequence (CDS) of *Btg2* gene was amplified from mouse cDNA by PCR and inserted into the pCI-neo vector using restriction enzyme digestion and ligation to generate the Btg2 overexpression vector (oe-Btg2). Using primers containing the Flag sequence, a Flag-tagged Btg2 overexpression vector (oe-Btg2-Flag) was constructed.

Construction of psiCHECK-2 vectors: The 3′ untranslated region (3′UTR) of *Btg2* gene was amplified from mouse genomic DNA by PCR and cloned into the multiple cloning site downstream of the Renilla luciferase gene in the psiCHECK-2 vector, yielding psi-wt-Btg2. Fusion PCR was performed to mutate the miR-146b-5p binding site within the *Btg2* 3′UTR of psi-wt-Btg2, generating psi-mut-Btg2.

### 2.7. Dual-Luciferase Reporter Assay

HEK293T cells were co-transfected with miR-146b-5p mimics and psiCHECK-2 vectors using Lipo6000™ (Beyotime, Beijing, China) transfection reagent in 12-well plates. The vectors were either psi-wt-Btg2 or psi-mut-Btg2, and the nucleic acids included a negative control and miR-146b-5p mimics. For each well, 500 ng of vector, 40 pmol of nucleic acid, and 1 μL of transfection reagent were used, and the transfection lasted for 24 h. After 24 h, luciferase activities were measured with the Dual-Luciferase Reporter Assay System (Promega, Madison, WI, USA). Cells were collected and thoroughly lysed in 1× Passive Lysis Buffer (Promega, Madison, WI, USA). Firefly luciferase activity and Renilla luciferase activity were then determined using the Luciferase Assay Reagent II and Stop & Glo^®^ reagent (Promega, Madison, WI, USA), respectively. Firefly luciferase activity served as an internal control, and the ratio of Renilla luciferase activity to Firefly luciferase activity was calculated.

### 2.8. EdU Staining

Coverslips were placed in 6-well plates before cell seeding. Transfection was performed when cells reached 50% confluence. EdU (Beyotime, Beijing, China) labeling was carried out at 34 h post-transfection. After adding EdU, cells were incubated for an additional 2 h to complete the labeling. Cells were then fixed with 4% paraformaldehyde overnight at 4 °C, followed by permeabilization with methanol and PBS containing 0.3% Triton X-100 (Beyotime, Beijing, China). A Click reaction mixture was prepared and incubated with the cells for 30 min. Finally, nuclei were counterstained with DAPI. EdU-positive cells were counted in a blinded manner with respect to group allocation.

### 2.9. Sequencing

Tissue transcriptome sequencing: Four biological replicates per group (*n* = 4 per time point) were selected for transcriptome sequencing. This sample size is consistent with established practices in similar mouse silicosis model transcriptome studies and represents a balance between achieving sufficient statistical power [24] and adhering to the 3R principle (reduction in animal usage). Sequencing was performed in a blinded manner with respect to group allocation. Total RNA was extracted from samples. RNA concentration and purity were measured using a NanoDrop 2000 (Thermo Scientific, Wilmington, DE, USA), and RQN values were determined with an Agilent 5300 (Agilent Technologies, Santa Clara, CA, USA). Qualified total RNA was subjected to mRNA enrichment using Oligo (dT) magnetic beads (Beyotime, Beijing, China), followed by random fragmentation to approximately 300 bp. Double-stranded cDNA was synthesized by reverse transcription, and after end repair, adapter ligation, fragment selection, and PCR enrichment, an Illumina strand-specific mRNA library was constructed. Paired-end 150 bp (PE150) sequencing was performed on a DNBSEQ platform (MGI, Shenzhen, China). Raw data were quality-controlled and filtered using fastp software (version v0.23.4) to remove low-quality reads, adapter contamination, and short sequences, yielding clean reads. Reads were aligned to the reference genome using HISAT2 (version v2.2.1), and gene expression was quantified with RSEM (version v1.3.3).

Tissue miRNA sequencing: Three biological replicates per group (*n* = 3 per time point) were selected for miRNA sequencing. Total RNA was extracted from samples. Small RNA fragments of 18–30 nt were enriched, followed by sequential ligation of the 3′ and 5′ adapters. cDNA was generated by reverse transcription and amplified by PCR to construct a small RNA library. After quality inspection, the library was sequenced on an Illumina NovaSeq platform (Illumina, San Diego, CA, USA) using single-end 50 bp (SE50) reads. Raw sequencing data were first subjected to quality control to remove low-quality sequences, adapter contamination, and overly short reads, obtaining clean reads. The clean reads were then aligned to the reference genome and the miRBase database (https://www.mirbase.org/, accessed on 15 May 2023) using Bowtie software (version v2.5.1). Based on expression quantification, differential expression analysis was performed using DESeq2 (version v1.38) to identify differentially expressed miRNAs.

Cell transcriptome sequencing: NIH3T3 cells were collected using TRIzol (Takara Biotechnology (Dalian) Co., Ltd., Dalian, China) at 36 h post-transfection, and transcriptome sequencing was conducted by Annoroad Gene Technology (Beijing) Co., Ltd. (Beijing, China). The general procedure was as follows: after total RNA extraction, concentration and integrity were assessed. Qualified samples were used to construct a eukaryotic reference transcriptome library, followed by PE150 sequencing on a DNBSEQ platform (MGI, Shenzhen, China) to obtain raw sequencing data. Data filtering was performed with fastp (version v0.23.4) to obtain high-quality clean reads. Sequence alignment was completed with HISAT2 (version v2.2.1), and raw read counts were obtained using featureCounts (Subread package version v2.1.1).

### 2.10. Co-IP and IP-MS

When cells reached approximately 60% confluence, they were transfected with Btg2 overexpression vectors (the oe-Btg2 vector or the oe-Btg2-Flag vector). At 36 h post-transfection, cells were lysed thoroughly on ice for 30 min using IP lysis buffer, and the supernatant (total protein) was collected by centrifugation. Before pre-clearing, a portion of the lysate was saved as input. After pre-clearing with Pierce Protein A/G Magnetic Beads to remove nonspecific binding proteins, the lysate was incubated with the appropriate antibodies: anti-Btg2 antibody (1:50) (22339-1-AP, Proteintech, Wuhan, China) or control IgG (1:50) (A7016, Beyotime, Beijing, China) for cells overexpressing Btg2, and anti-Flag antibody (1:50) (14793S, Cell Signaling Technology, Danvers, MA, USA) or IgG for cells overexpressing Btg2-Flag. All antibodies used in this study were commercial products, and their suitability for immunoprecipitation (IP) has been validated by the suppliers. The mixtures were incubated at 4 °C overnight to allow immune complex formation. Immune complexes were then captured with Pierce Protein A/G Magnetic Beads, eluted, and analyzed by Western blot or mass spectrometry.

Mass spectrometry analysis was performed as follows. The peptide mixture was first separated by liquid chromatography to reduce sample complexity, then peptides were converted into gas-phase ions via electrospray ionization (ESI) (Thermo Scientific, Waltham, MA, USA). Finally, the ions entered the mass analyzer (Thermo Scientific, Waltham, MA, USA) and were separated and detected according to their mass-to-charge ratio (*m*/*z*). For mass spectrometry data analysis, proteins identified in the IgG isotype control group were defined as non-specific binding proteins and subtracted from the candidate interacting protein list of each experimental group. To further exclude false positives caused by antibody non-specificity, we performed two independent IP-MS assays using antibodies against different epitopes (anti-Btg2 antibody targeting the Btg2 protein itself, and anti-Flag antibody targeting the C-terminal Flag tag). Proteins commonly identified in both antibody pull-down groups after IgG background subtraction were considered high-confidence specific interacting partners of Btg2.

### 2.11. Bioinformatics Analysis

Time-series clustering (K-means): Genes with low expression were filtered out, retaining those with TPM > 1 in at least 20% of the samples. For each gene, the average TPM value within each time group was calculated, and the resulting mean expression values across the five time points were Z-score normalized (mean = 0, standard deviation = 1) to eliminate differences in absolute expression levels among genes. To determine the optimal number of clusters, we performed the elbow method by calculating the within-cluster sum of squares (WSS) for k values ranging from 2 to 10. The elbow point was identified at k = 7. K-means clustering was then applied to the normalized time-series expression matrix, with each gene assigned to the nearest cluster center. The centroid curve of each cluster was defined as the average normalized expression value of all genes in that cluster at each time point. Based on the seven gene clusters (Clusters 1–7) obtained from time-series clustering, GO biological process (BP) enrichment analysis was performed for the genes within each cluster. The Gene Ontology database (https://geneontology.org/, accessed on 20 May 2025) was used for functional annotation.

K-means clustering of gene expression profiles after miR-146b-5p mimic transfection: K-means clustering was performed on the top 2000 most variable genes in NIH3T3 cells transfected with miR-146b-5p mimics or negative control. The optimal number of clusters was determined by the silhouette method (k = 2). GO biological process enrichment analysis was conducted for the genes in each cluster. The top 20 most significant GO terms in Cluster 1 (ranked by padj) were selected, and their enrichment significance in Cluster 2 was also evaluated.

Analysis of pathway-level mean log_2_(fold change) of differentially expressed proteins induced by Myh10 knockout: The pathway-level analysis was performed based on log2-transformed ratios of differentially expressed proteins. The raw proteomic data for Myh10 knockout fibroblasts were obtained from the Supplementary Materials of Kim et al. [25] (available via the PRIDE repository, dataset identifier PXD011139, https://www.ebi.ac.uk/pride/archive/projects/PXD011139, accessed on 2 March 2026). We re-analyzed the raw protein expression data to calculate log2-transformed ratios (Myh10-knockout/control) for each protein across three biological replicates. Mean log2-transformed ratio of each gene across three biological replicates was first calculated to obtain gene-level mean log2-transformed ratios. Gene sets were derived from the mouse Hallmark gene sets in the MSigDB database (50 pathways in total, https://www.gsea-msigdb.org/gsea/msigdb, accessed on 2 March 2026). For each pathway, its gene symbols were extracted and intersected with the list of quantified genes, retaining pathways containing at least three quantified genes. The mean of gene-level mean log2-transformed ratios within each pathway was calculated as the representative value for that pathway. All pathways were sorted in descending order by the absolute value of the mean log2-transformed ratio, and the top 15 pathways with the largest changes were selected and then re-ordered from the highest to the lowest mean log2-transformed ratio. A bar plot was generated to visualize the mean log2-transformed ratio of each pathway, with up-regulation (positive values) shown in red and down-regulation (negative values) in blue. The number of quantified genes used for calculation in each pathway (*n*) was annotated next to the bar.

The effect of Prrx2 overexpression on Hallmark pathways was analyzed by ssGSEA: A previous study performed transcriptome sequencing after Prrx2 overexpression [26]. The raw RNA-seq data from Rodriguez et al. [26] were obtained from the Gene Expression Omnibus (GEO) (https://www.ncbi.nlm.nih.gov/geo/query/acc.cgi?acc=GSE199538, accessed on 8 March 2026) under accession number GSE199538. We re-analyzed the raw count data to perform ssGSEA analysis. Raw counts were filtered using edgeR (version v4.8.2, retaining genes with CPM ≥ 10 in at least three samples) and log_2_(CPM + 1) was calculated as the expression matrix. Gene sets were obtained from all human Hallmark gene sets (50 pathways) in the MSigDB database (https://www.gsea-msigdb.org/gsea/msigdb, accessed on 8 March 2026). For each pathway, its Ensembl gene IDs were extracted and intersected with genes present in the expression matrix, retaining pathways containing at least five genes. The escape.matrix function from the escape package (version v2.6.1) was used to compute ssGSEA enrichment scores for each sample and each pathway. The 15 pathways with the smallest padj were selected for heatmap visualization. Row Z-score normalization was applied to the enrichment scores of each pathway in the heatmap.

Single-cell database analysis: The cell-type-specific expression pattern of Btg2 in human pulmonary fibrosis was analyzed using the Idiopathic Pulmonary Fibrosis (IPF) Cell Atlas database (https://www.ipfcellatlas.com/, accessed on 2 November 2024). The publicly available dataset from Kaminski/Rosas lab (https://www.ncbi.nlm.nih.gov/geo/query/acc.cgi?acc=GSE136831, accessed on 2 November 2024) was adopted for analysis, which contains single-cell RNA-seq profiles of lung tissues from 3 patients with IPF and 3 age-matched healthy donors, with a total of ~92,000 single cells captured and annotated. Cells in the dataset are classified into three major lineages: Epithelial, Immune and Stromal. UMAP dimensionality reduction visualization was generated through the online analysis tool to display the distribution of Btg2 expression across stromal cell subsets.

Differential expression analysis in human lung fibroblasts: The expression difference in BTG2 between normal and fibrotic human lung fibroblasts was analyzed using the Gene Expression tool from the CZ CELLxGENE platform (also referred to as the GENE Discover online database, https://cellxgene.cziscience.com/gene-expression (accessed on 10 April 2026). The integrated human pulmonary fibrosis single-cell dataset curated by the platform was used for analysis. Cell type was filtered to lung fibroblasts, and cells were grouped by disease status (normal vs. pulmonary fibrosis). The analysis included ~101,500 cells from normal lung tissues and ~6600 cells from pulmonary fibrosis lung tissues. The expression distribution of BTG2 in the two groups was visualized for comparative analysis.

### 2.12. Graphical Abstract

The Graphical abstract was created with BioGDP [27].

### 2.13. Data Analysis

For cell-based functional assays (including RT-qPCR, Western blot, CCK-8, EdU, and dual-luciferase reporter assays), three independent biological replicates (*n* = 3) were performed, which is the standard practice in cell and molecular biology research to ensure reproducibility. Results are presented as mean ± SEM. Prior to statistical comparison, normality was assessed using the Shapiro–Wilk test, and homogeneity of variance was assessed using the F-test. When both assumptions were met, Student’s *t*-test was used for comparisons between two groups. When the assumption of equal variance was violated, Welch’s corrected *t*-test was applied. Exact *p*-values for all statistical comparisons are provided in Supplementary Table S1. A *p*-value < 0.05 was considered statistically significant (* *p* < 0.05, ** *p* < 0.01).

## 3. Results

### 3.1. Screening of Key miRNAs and Key Genes During Pulmonary Fibrosis

To investigate the key regulatory mechanisms of fibroblasts during pulmonary fibrosis, mice were exposed to silica dust by dynamic inhalation for 7, 14, 28, and 56 days. To evaluate the effect of dust exposure on lung tissue, H&E staining was performed after the exposure period (Figure 1A). H&E staining showed that, compared with control mice, widened pulmonary interstitium was observed at 28 days of dust exposure, and increased abnormal alveoli and aggravated fibrosis were evident at 56 days.

To characterize the key regulatory mechanisms at both transcriptional and post-transcriptional levels during pulmonary fibrosis, we performed transcriptome (Supplementary Table S2) and miRNA (Supplementary Table S3) sequencing on lung tissues from mice exposed to silica dust for 7, 14, 28, and 56 days. Conventional differential expression analysis (e.g., identifying differentially expressed genes at each time point) only captures expression changes at individual time points and fails to reflect dynamic alterations over a continuous time course. Given that our data represent longitudinal expression profiles across multiple time points (0, 7, 14, 28, and 56 days), we employed time-series clustering rather than traditional differentially expressed gene analysis, allowing a better characterization of temporal gene expression patterns throughout disease progression. After filtering the TPM matrix, Z-score normalization was applied, and K-means clustering (k = 7) was used to partition the genes into seven clusters with distinct temporal expression patterns (Figure S1). Subsequently, GO enrichment analysis was performed for each cluster, and the top five most significant GO terms per cluster were selected to generate a bubble chart (Figure 1B). The clusters displayed clearly distinct functional themes corresponding to sequential pathological stages of silicosis. Early-stage responses (Clusters 5 and 7) were mainly enriched for ciliary clearance (Cluster 5) as well as energy metabolism and ER stress (Cluster 7), reflecting the immediate cellular stress triggered by silica particle deposition. Cluster 2 represented the early-to-intermediate transition phase, and was predominantly characterized by sustained ER stress and protein homeostasis regulation. Intermediate-stage responses (Clusters 3 and 4) were dominated by innate and adaptive immune inflammatory activation. Late-stage responses (Clusters 1 and 6) were driven by TGF-β-mediated fibrotic remodeling (Cluster 1) and vascular remodeling (Cluster 6), consistent with the establishment and progression of chronic fibrosis.

After confirming that the model effectively recapitulated the progression of silicosis, we performed miRNA sequencing and principal component analysis (Figure 2A). Then, we screened differentially expressed miRNAs (padj < 0.05 and |log_2_(fold change)| > 1) at each time point using the miRNA sequencing data (Figure 2B). Venn diagrams were then employed to compare the upregulated (Figure 2C) and downregulated (Figure 2D) differentially expressed miRNAs across the time points. Given the H&E staining results, which revealed pathological changes at 28 and 56 days, we focused on the differentially expressed miRNAs induced at these two time points to evaluate their potential involvement in fibroblast activation and proliferation during fibrosis. Six miRNAs were commonly upregulated at both 28 and 56 days of exposure (mmu-miR-146b-5p, mmu-miR-146b-3p, mmu-miR-96-5p, mmu-miR-132-3p, mmu-miR-338-3p, mmu-miR-708-3p), and four were commonly downregulated (mmu-miR-486b-5p, mmu-miR-486a-5p, mmu-miR-1941-5p, mmu-miR-676-3p).

miRNA function is closely linked to the regulation of its target genes. We therefore predicted the target genes of these miRNAs using the TargetScanMouse 8.0 database (Supplementary Table S4), aiming to establish the miRNA–target regulatory relationships. We then asked whether any of the differentially expressed genes (DEGs) shared between 28 and 56 days of exposure were among the predicted targets of the 10 differentially expressed miRNAs. Analysis of the shared DEGs identified 102 upregulated and 46 downregulated genes (Figure 2C,D). By integrating the target predictions, we used a Sankey diagram to depict the relationships between the miRNAs and the genes that were both DEGs and predicted targets (Figure 2C,D and Supplementary Table S5). Notably, the downregulated gene *Btg2* emerged as the most extensively regulated hub gene, potentially targeted by three upregulated miRNAs: miR-132-3p, miR-146b-5p, and miR-146b-3p. To further validate the regulatory relationships between these miRNAs and *Btg2*, we performed cross-validation using two independent target prediction databases, miRDB and miRWalk. Among these three miRNAs, only miR-146b-5p was consistently predicted to suppress *Btg2* expression across all three databases (TargetScanMouse 8.0, miRDB (Mouse), and miRWalk (Mouse)).

We next asked whether Btg2 is associated with fibroblasts by first examining its expression localization in lung tissues using the Idiopathic Pulmonary Fibrosis (IPF) Cell Atlas database (Figure 2E). The results revealed that *BTG2* was highly expressed specifically in fibroblasts. We then explored the relationship between Btg2 and pulmonary fibrosis by analyzing its expression in normal versus fibrotic lung tissues using the GENE Discover online database (Figure 2F). Compared with fibroblasts from normal lungs, *BTG2* expression was downregulated in fibroblasts from fibrotic lungs, consistent with the downregulation observed in our sequencing data after SiO_2_ exposure. We therefore selected *Btg2* gene for subsequent functional studies in fibroblasts.

### 3.2. miR-146b-5p Promotes Fibroblast Proliferation at Least Partially by Targeting Btg2

Among the three miRNAs that target *Btg2*, miR-146b-5p was unique in that *Btg2* was its sole predicted target among the DEGs. Moreover, cross-validation using miRDB and miRWalk confirmed that miR-146b-5p, but not the other two miRNAs, was consistently predicted to target *Btg2* across all three independent databases. We therefore selected miR-146b-5p for subsequent studies to minimize interference from other target genes. We first examined miR-146b-5p expression in SiO_2_-exposed mouse lung tissues by RT-qPCR (Figure 3A). The results showed that miR-146b-5p was significantly upregulated in the lung tissues of mice exposed to dynamic inhalation of silica dust for 28 days. We next asked whether this upregulation represented background noise or a positive response to fibrosis, and thus treated NIH-3T3 fibroblasts with TGF-β and measured miR-146b-5p expression (Figure 3B). TGF-β stimulation induced miR-146b-5p upregulation. Collectively, miR-146b-5p expression was significantly altered both in vivo and in vitro.

We transfected NIH3T3 cells with miR-146b-5p mimics (Figure 3C) and subsequently performed transcriptome sequencing (Supplementary Table S6). Given that time-series clustering (K-means) of the transcriptome data from SiO_2_-exposed mice effectively captured the progression of silicosis, we applied the same clustering approach to the transcriptome data obtained after mimic transfection. K-means clustering partitioned the transcriptomes of miR-146b-5p-overexpressing and control fibroblasts into two distinct clusters. Among the top 20 GO BP terms enriched in Cluster 1, multiple terms pointed to “mitotic chromosome segregation” (Figure 3D). This result suggests that elevated miR-146b-5p expression may affect mitosis and the cell cycle in fibroblasts.

*Btg2*, a predicted target of miR-146b-5p, has been implicated in cell cycle regulation and proliferation in various cell types. Thus, miR-146b-5p may regulate the cell cycle through Btg2. We next investigated the regulatory effect of miR-146b-5p on Btg2. After overexpressing miR-146b-5p mimics, RT-qPCR analysis revealed that miR-146b-5p suppressed *Btg2* expression (Figure 3E). We then analyzed the 3′UTR of *Btg2* and the miR-146b-5p complementary site. By constructing a phylogenetic tree, we first examined the 3′UTR of *Btg2* across multiple species (Figure 3F). The phylogenetic tree constructed based on the 3′UTR is consistent with the morphological classification. Analysis of the binding site revealed that this site is present in primates and rodents but absent in cattle and dogs (Figure 3F).

We next verified whether miR-146b-5p directly regulates *Btg2* using the psiCHECK-2 vector. The 3′UTR of *Btg2* containing the predicted miR-146b-5p binding site was cloned into psiCHECK-2 (psi-wt-Btg2), and a mutant construct with the binding site disrupted was also generated (psi-mut-Btg2) (Figure 3G). Co-transfection of miR-146b-5p mimics with psi-wt-Btg2 into HEK293T cells for 24 h significantly reduced the relative Renilla luciferase activity, whereas co-transfection of miR-146b-5p mimics with psi-mut-Btg2 did not alter the relative luciferase activity (Figure 3H). These results demonstrate that miR-146b-5p directly targets the complementary site in the 3′UTR of *Btg2* and exerts a direct inhibitory effect on *Btg2* expression through this site.

### 3.3. Btg2 Suppresses Fibroblast Proliferation and May Affect the G1/S Transition

We next examined the function of Btg2. First, we measured *Btg2* expression by RT-qPCR in the lung tissues of mice exposed to silica dust via dynamic inhalation (Figure 4A) and in fibroblasts stimulated with TGF-β (Figure 4B). *Btg2* was significantly downregulated under both conditions. We then designed a small interfering RNA targeting Btg2 and constructed a Btg2 overexpression (oe-Btg2) vector, and transfected them into NIH3T3 cells. After transfection, we confirmed *Btg2* expression at the mRNA level (Figure S2) and protein level (Figure 4C,D). These results demonstrated effective modulation of Btg2 expression by these approaches.

We sought to characterize the function of Btg2 in fibroblasts by performing transcriptome sequencing after Btg2 knockdown (Supplementary Table S6) and overexpression (Supplementary Table S7). Genes exhibiting opposite directions of change in these two conditions were identified and subjected to GO BP enrichment analysis. Multiple significantly enriched GO terms associated with cell proliferation were identified, and the top 10 proliferation-related terms are shown in Figure 4E. These terms included smooth muscle cell proliferation, endothelial cell proliferation, muscle cell proliferation, regulation of cell growth, and the G1/S transition of the mitotic cell cycle. These findings indicate that Btg2 downstream genes are broadly involved in cell cycle and proliferation control, consistent with a mechanism in which miR-146b-5p targets and inhibits Btg2, thereby facilitating mitotic chromosome segregation and cell cycle progression in fibroblasts.

We next experimentally examined whether Btg2 affects fibroblast proliferation. First, after inhibiting Btg2 with siRNA or overexpressing Btg2 using an oe-Btg2 vector, cell viability was measured by CCK-8 assay. The results showed that Btg2 knockdown enhanced cell viability (Figure 5A), whereas Btg2 overexpression reduced fibroblast viability (Figure 5B). The enrichment analysis indicated that Btg2 affects mitotic chromosome segregation and the G1/S transition. The correlation between Btg2 and the cell cycle was further investigated by EdU labeling. Upon Btg2 knockdown, more cells entered S phase (Figure 5C, Supplementary Table S8). Conversely, when Btg2 was overexpressed, fewer cells entered S phase (Figure 5D and Supplementary Table S8). Consistent with the enrichment of G1/S transition-related gene sets in the transcriptome, EdU assay results confirmed that Btg2 negatively regulates fibroblast entry into S phase. However, the EdU incorporation assay only reflects the proportion of cells undergoing active DNA synthesis and cannot distinguish whether this effect is caused by G1/S transition arrest or by indirect effects of alterations in other cell cycle phases.

### 3.4. Btg2 Regulates Proliferation via Prrx2 and Myh10 Interactions

The molecular mechanism by which Btg2 regulates the cell cycle in fibroblasts was investigated. A Btg2 overexpression vector carrying a C-terminal Flag tag (oe-Btg2-Flag) was constructed based on the oe-Btg2 vector. After overexpression of the oe-Btg2 and oe-Btg2-Flag vectors in NIH3T3 cells, IP-MS was performed using anti-Btg2 (Supplementary Table S9) and anti-Flag antibodies (Supplementary Table S10), respectively. To identify specific interacting proteins, we first subtracted all proteins identified in the IgG isotype control from each IP-MS dataset to remove non-specific background binding (Figure 6A). We then intersected the candidate protein lists from the anti-Btg2 and anti-Flag IP-MS assays to further eliminate false positives arising from antibody-specific non-specific binding. A total of 8 proteins were consistently identified as Btg2-interacting candidates in both independent pull-down assays, among which Myh10 and Prrx2 were the top two most abundant proteins (Figure 6A). The interactions of Btg2 with Prrx2 and Myh10 were verified by co-IP, followed by Western blot analysis (Figure 6B).

We investigated whether Myh10 and Prrx2 are also involved in cell cycle regulation. A previously published study knocked out Myh10 in fibroblasts and performed proteomic sequencing [25]. Based on differentially expressed proteins, we calculated the mean log2-transformed ratio (Myh10-knockout/control) of genes within each Hallmark pathway and selected the top 15 pathways by the absolute value of change (Figure 6C). The results showed that in Myh10-knockout fibroblasts, multiple pathways related to mitosis and cell cycle were enriched among the top 15.

A previous study performed transcriptome sequencing after Prrx2 overexpression [26]. Based on the RNA-seq data, we conducted ssGSEA analysis on Hallmark pathways and selected the top 15 pathways by statistical significance. The results showed that the top 15 pathways included multiple pathways closely related to chromosome segregation, cell cycle regulation, and proliferation (Figure 6D). Thus, Btg2 may participate in cell cycle regulation through its interaction with Myh10 and Prrx2.

## 4. Discussion

The core pathological process of pulmonary fibrosis is the abnormal activation of fibroblasts and excessive deposition of extracellular matrix [28]. Non-coding RNAs, as important regulators of gene expression [29,30], play significant roles in fibrotic diseases [31]. We systematically characterized the dynamic changes in gene expression during SiO_2_-induced mouse pulmonary fibrosis by integrating time-series transcriptome and miRNA sequencing. miR-146b-5p was significantly upregulated and directly targeted the 3′UTR of *Btg2* to suppress its expression, thereby promoting fibroblast proliferation. Mechanistically, Btg2 was found to interact with Prrx2 and Myh10.

A study on intermittent hypoxia stress in mice across multiple time points found that the enrichment results of differentially expressed genes at each individual time point did not include pathways associated with disease progression [32]. However, when genes correlated with the time of stress were extracted for enrichment analysis, multiple pathways related to injury and neurodegeneration were enriched. In our study, we identified multiple clusters through time-series clustering, and the enrichment results of these clusters were highly consistent with the temporal events of the SiO_2_ pathogenic process. These findings provide insights for the analysis of multi-time-point stress data.

During the study, we validated the direct regulatory effect of miR-146b-5p on *Btg2*. Btg2 is known to inhibit proliferation by blocking the G1/S transition [33,34]. In fibroblasts, we found that Btg2 knockdown promoted fibroblast entry into S phase, whereas Btg2 overexpression produced the opposite effect, suggesting that miR-146b-5p relieves cell cycle arrest by downregulating *Btg2*, thereby promoting proliferation. Currently, our results are derived mainly from a mouse SiO_2_ exposure model and the NIH3T3 fibroblast cell line, which differ from various types of human pulmonary fibrosis. Notably, we have not performed rescue experiments to confirm that the pro-proliferative effect of miR-146b-5p is specifically mediated through *Btg2*, nor have we carried out in vivo functional validation such as pulmonary delivery of miR-146b-5p antagomir or Btg2 overexpression vectors. In addition, the mechanistic validation was performed in NIH3T3 cells (mouse embryonic fibroblasts), which are not of lung origin, and the dual-luciferase reporter assay was conducted in HEK293T cells. Thus, the direct applicability of these findings to human lung fibroblasts and pulmonary fibrosis is limited. Therefore, the expression changes and regulatory relationship of miR-146b-5p and BTG2 in human pulmonary fibrosis remain to be validated, and its potential as a therapeutic target requires further investigation in models that better mimic the clinical setting.

Using IP-MS combined with Co-IP, we found that Btg2 interacts with Myh10 and Prrx2. Previous studies have largely focused on the functions of Myh10 in epithelial–mesenchymal transition [35] and extracellular matrix deposition [25], as well as the roles of Prrx2 in tumor metastasis [36] and cell differentiation [37]. By integrating public database analysis and GSEA, we found that both Myh10 and Prrx2 are associated with cell proliferation. However, the functional relevance of these interactions to fibroblast proliferation is mainly inferred through pathway enrichment analyses of published datasets [25,26]. Definitive causal relationships (e.g., epistasis experiments knocking down Prrx2 or Myh10 in the context of regulating Btg2) were not validated in our results. Therefore, whether Btg2 directly affects fibroblast proliferation through Myh10 and Prrx2 remains unclear. Further functional studies, including epistasis experiments, are needed to elucidate the detailed mechanisms underlying this interaction.

This study used only male mice. Although the selection of male mice was based on the epidemiological feature of a higher prevalence of silicosis in males, whether the miR-146b-5p/*Btg2* regulatory mechanism identified in this study also applies to females requires further validation. Furthermore, the inference regarding cell cycle arrest checkpoints in this study was primarily based on EdU incorporation assays and transcriptomic pathway analysis, and direct quantification of the proportion of cells in each cell cycle phase by propidium iodide staining and flow cytometry has not yet been performed. Therefore, the specific checkpoint at which Btg2 regulates the cell cycle remains to be further confirmed by dedicated cell cycle phase analysis experiments.

## 5. Conclusions

We established a mouse model of pulmonary fibrosis through dynamic dust inhalation, and integrated multi-omics sequencing, bioinformatics analysis, and experimental validation to reveal the regulatory role of miR-146b-5p and Btg2 in fibroblast proliferation. The interactions of Btg2 with Myh10 and Prrx2 were also preliminarily identified. These findings provide new theoretical knowledge and experimental evidence for the molecular mechanisms of silica-induced pulmonary fibrosis in a mouse model, although further studies in human samples are needed to confirm their translational relevance.

## Figures and Tables

**Figure 1:**
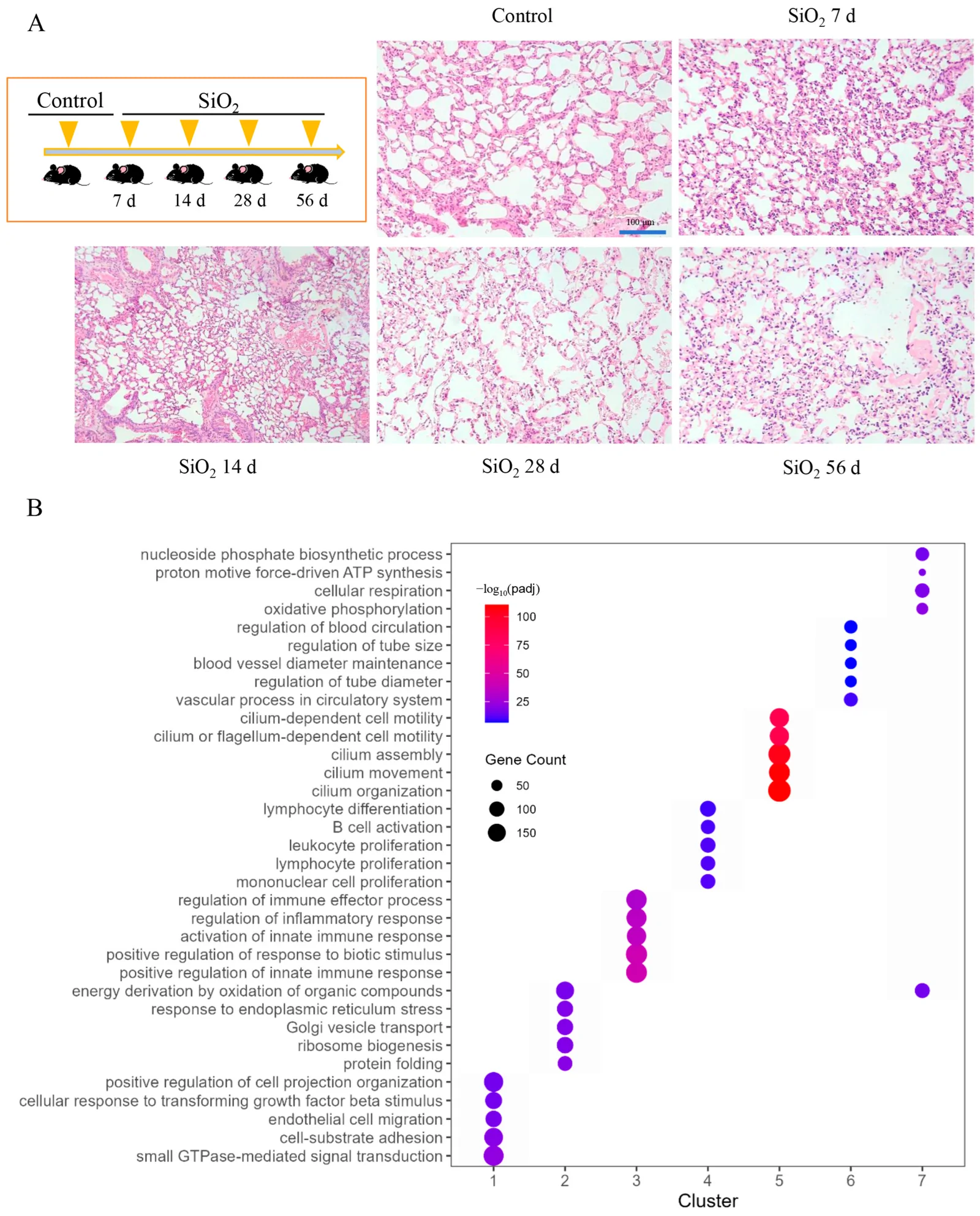
Pathological process of pulmonary fibrosis induced by SiO_2_ exposure. (**A**) H&E staining of lung tissue. H&E staining reveals pathological changes in the control group and at 7, 14, 28, and 56 days of exposure. Scale bar: 100 μm. (**B**) Bubble chart of the top five GO biological process terms for each temporal cluster (Clusters 1–7). The x-axis indicates cluster number, and the y-axis indicates GO terms.

**Figure 2:**
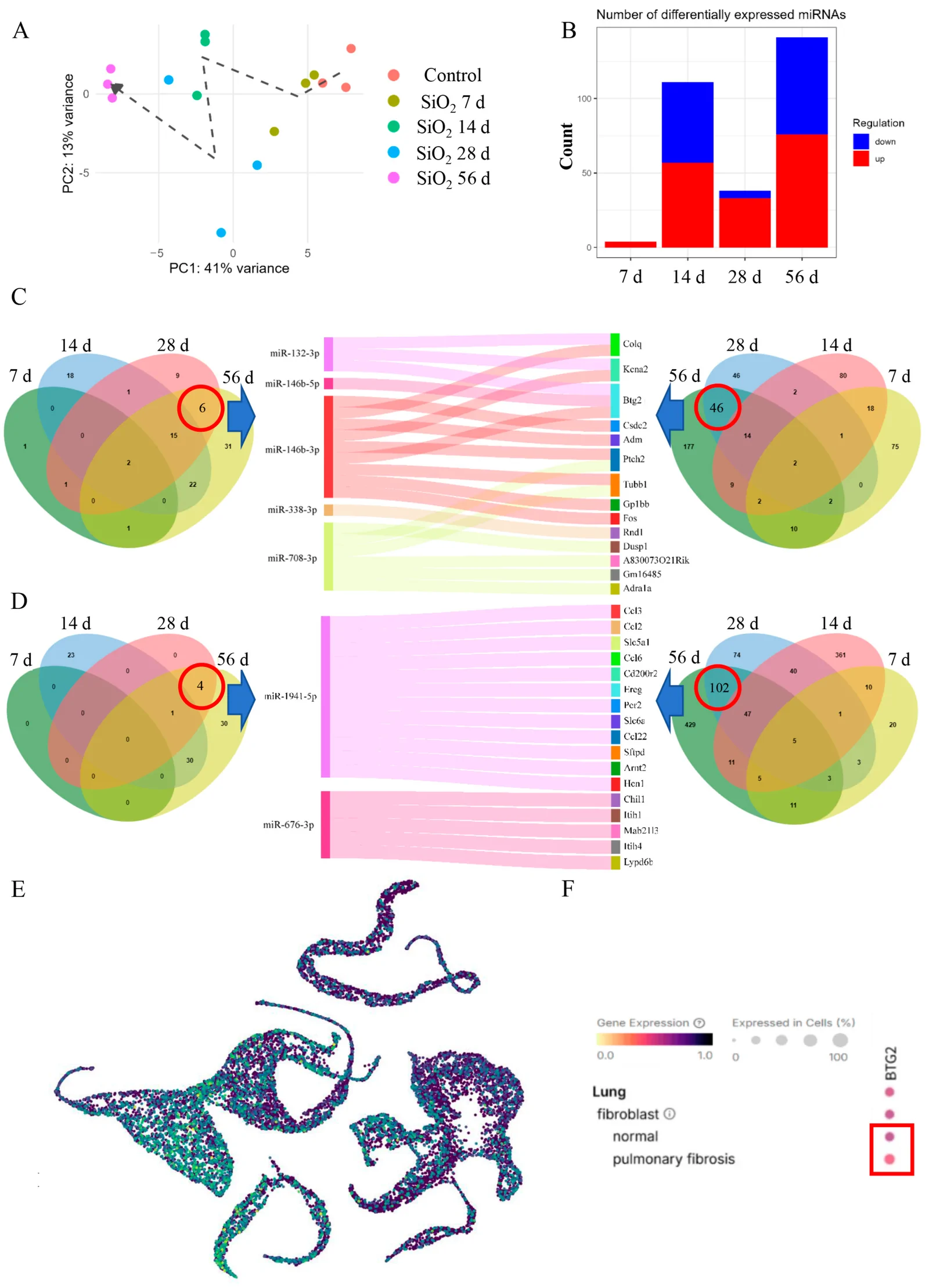
Identification of miRNAs and key downstream genes under SiO_2_ exposure. (**A**) PCA of miRNA expression profiles under different SiO_2_ treatment durations. (**B**) Statistical summary of differentially expressed miRNAs at different time points of SiO_2_ exposure. Red and blue represent upregulated and downregulated miRNAs, respectively. (**C**,**D**) Venn diagrams showing the overlap of upregulated and downregulated differentially expressed miRNAs, as well as differentially expressed genes, across the indicated time points (7, 14, 28, and 56 days). A Sankey diagram was also included to depict the relationships between key miRNAs and their potential target genes among DEGs at the corresponding time points. (**E**) Distribution of *BTG2* expression across various cell types in lung tissue based on the Idiopathic Pulmonary Fibrosis Cell Atlas database. (**F**) Comparison of *BTG2* expression between normal and fibrotic human lung fibroblasts analyzed using the CZ CELLxGENE (GENE Discover) database (fibroblast cells: ~101,500 normal cells, ~6600 pulmonary fibrosis cells). Color intensity corresponds to *BTG2* expression level, and circle size indicates the proportion of *BTG2*-expressing cells.

**Figure 3:**
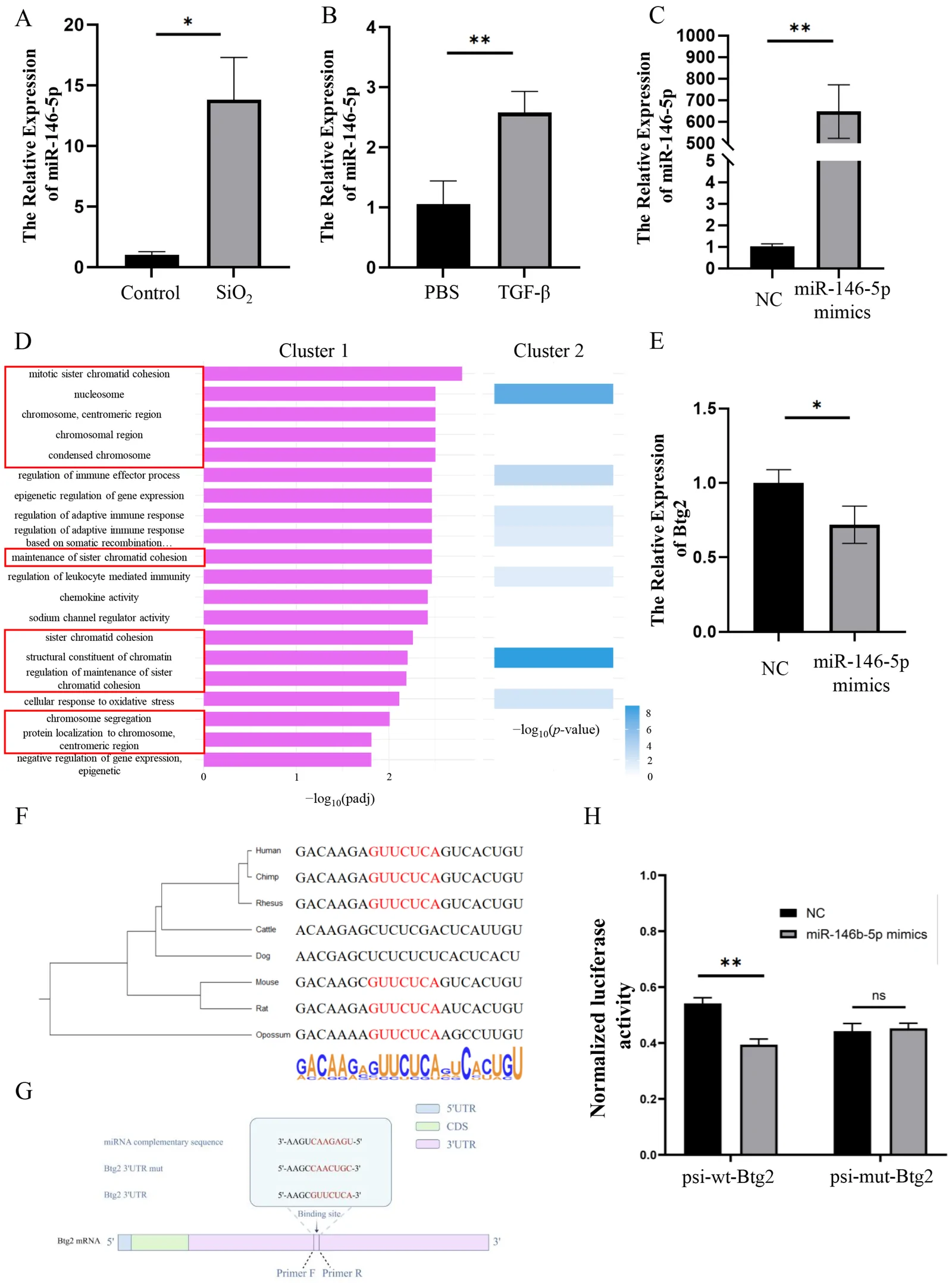
Functional study of miR-146b-5p in fibroblasts. (**A**) RT-qPCR validation of miR-146b-5p expression in mouse lung tissue after 28 days of SiO_2_ exposure. U6 served as an internal control. *: *p* < 0.05, *n* = 3. (**B**) RT-qPCR validation of miR-146b-5p expression after TGF-β stimulation. U6 served as an internal control. **: *p* < 0.01, *n* = 3. (**C**) RT-qPCR validation of miR-146b-5p overexpression after mimics transfection. U6 served as an internal control. **: *p* < 0.01, *n* = 3. (**D**) Comparative GO enrichment analysis of the two gene clusters derived from K-means clustering of miR-146b-5p-overexpressing and control fibroblasts. The top 20 most significantly enriched GO terms in Cluster 1, ranked by −log_10_(padj). Red squares indicate multiple terms pointing to “mitotic chromosome segregation”. (**E**) RT-qPCR analysis of *Btg2* expression after miR-146b-5p mimics transfection. *Gapdh* served as an internal control. *: *p* < 0.05, *n* = 3. (**F**) The phylogenetic tree was constructed based on the 3′UTR of *Btg2* across multiple species. The complementary sites of miR-146-5p and flanking sequences in the 3′UTR of *Btg2* of multiple species were analyzed. (**G**) Illustration of PCR amplification and mutation of the 3′UTR fragments of *Btg2*. (**H**) After co-transfection of miRNA mimics with recombinant vector (psi-wt-Btg2, psi-mut-Btg2) for 24 h, the activities of Renilla luciferase and Firefly luciferase were detected. **: *p* < 0.01, *n* = 3.

**Figure 4:**
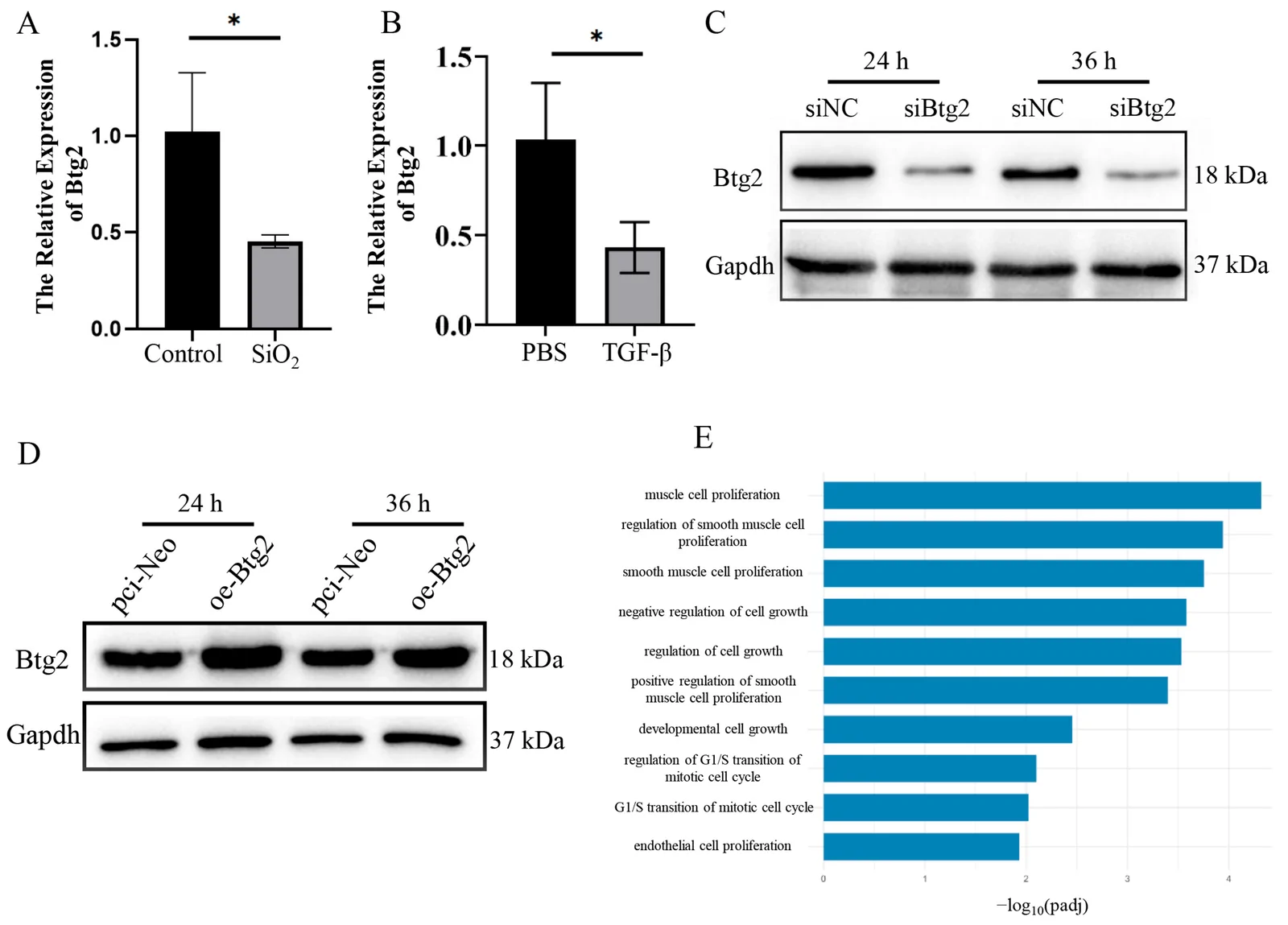
Transcriptome analysis of the regulatory role of Btg2 in fibroblast proliferation. (**A**) RT-qPCR validation of *Btg2* expression in mouse lung tissue after 28 days of SiO_2_ exposure by dynamic inhalation. *Gapdh* served as an internal control. *: *p* < 0.05, *n* = 3. (**B**) RT-qPCR validation of *Btg2* expression in fibroblasts after TGF-β stimulation. *Gapdh* served as an internal control. *: *p* < 0.05, *n* = 3. (**C**,**D**) Western blot analysis of Btg2 protein levels after transfection with Btg2 siRNA (**C**) or overexpression vector (**D**). (**E**) Proliferation-related GO terms enriched among genes oppositely regulated by Btg2 knockdown and overexpression. The bar plot shows the top 10 most significantly enriched terms, ranked by −log_10_(padj). The original western blot images can be found in Supplementary Materials.

**Figure 5:**
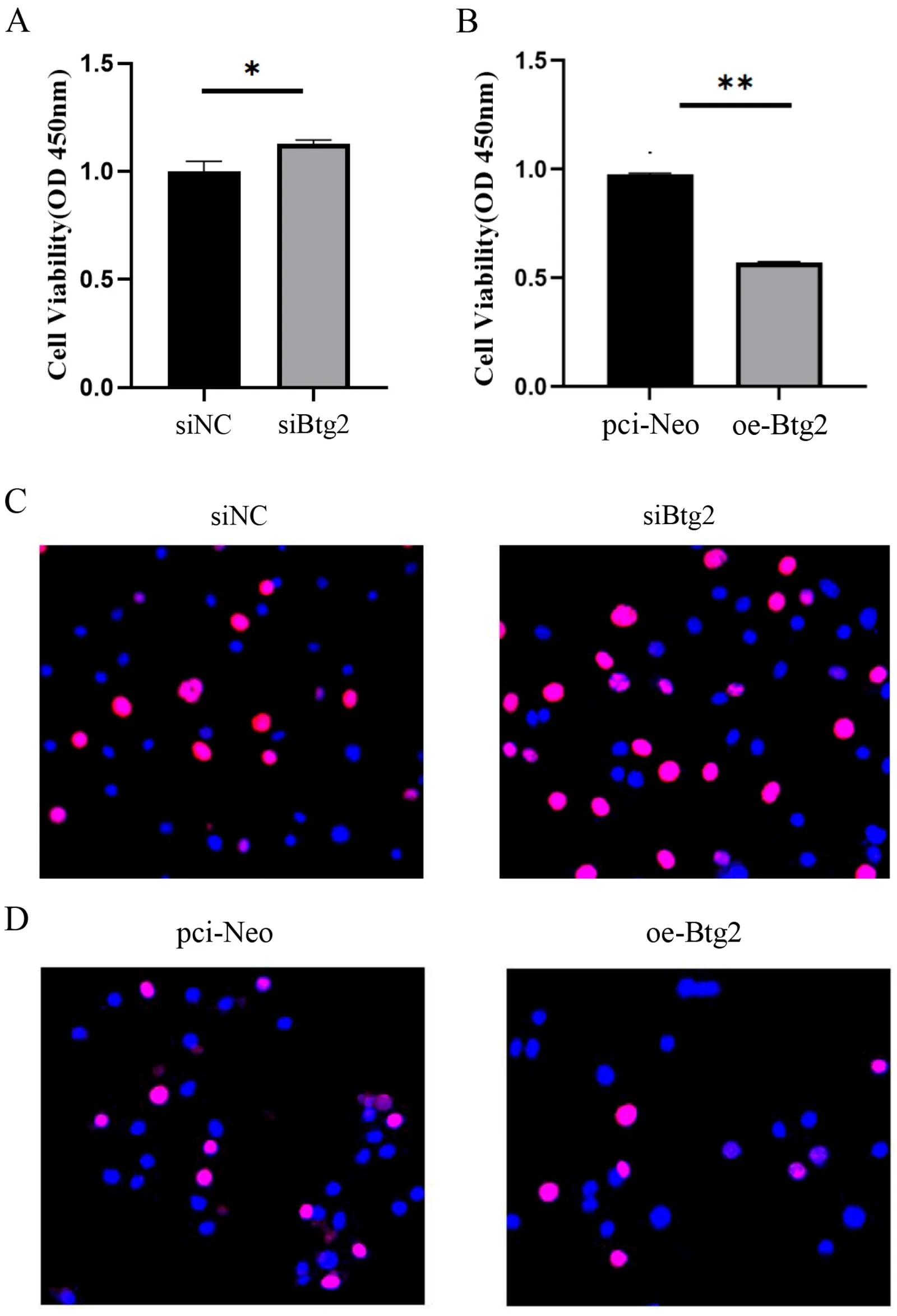
Btg2 affects the fibroblast cell cycle. (**A**,**B**) Cell viability was assessed using the CCK-8 assay after transfection with *Btg2* siRNA or oe-Btg2 vector. *: *p* < 0.05, **: *p* < 0.01, *n* = 3. (**C**,**D**) The effect on cell proliferation was examined using an EdU kit after transfection with *Btg2* siRNA or oe-Btg2 vector. Pink represents EdU staining; blue represents DAPI-stained nuclear DNA.

**Figure 6:**
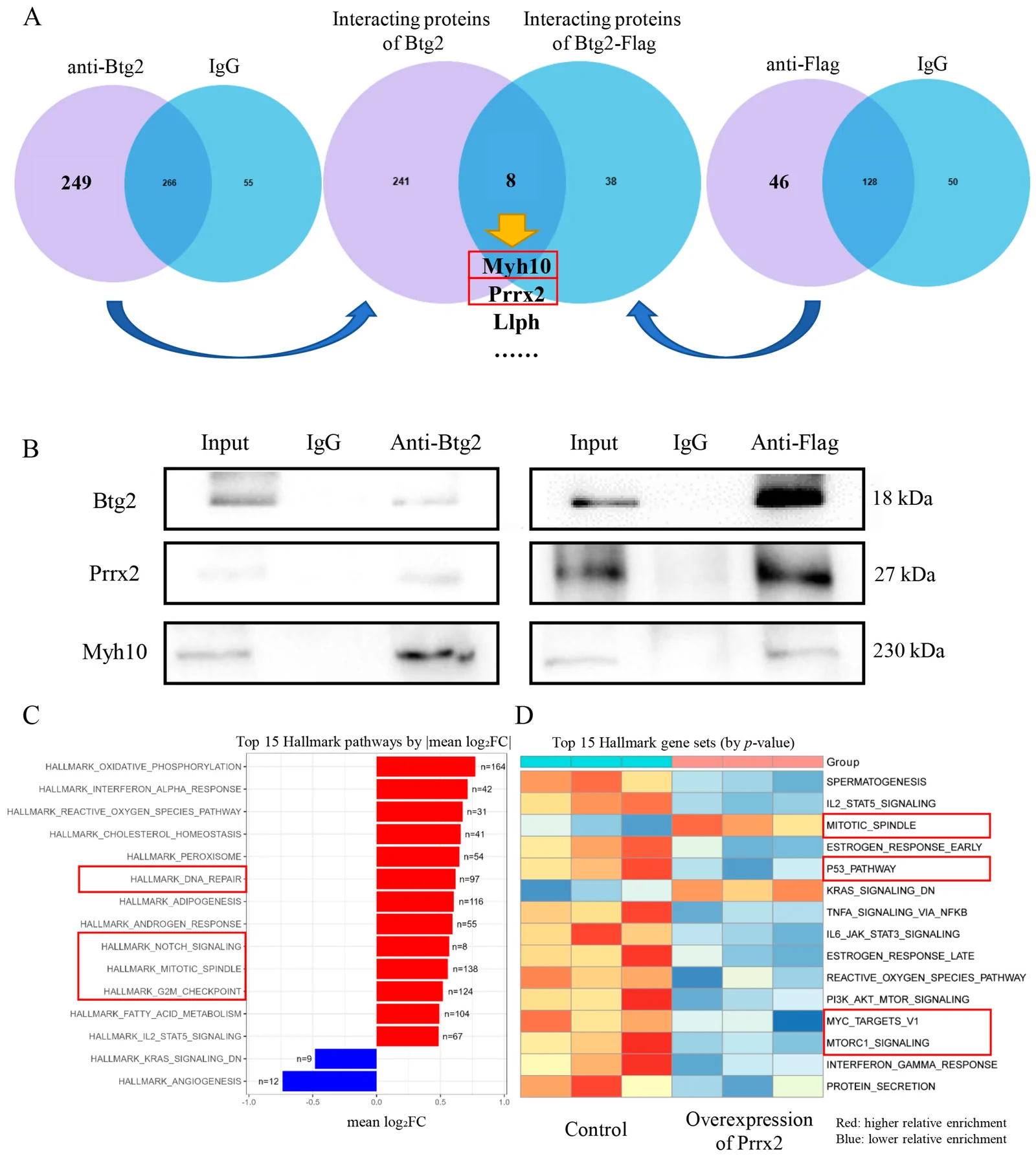
Screening of Btg2 interacting proteins. (**A**) Venn diagram showing the target proteins of Btg2 and Btg2-Flag identified by IP-MS. (**B**) Co-IP validation of the interactions between Btg2 and Myh10 or Prrx2. (**C**) Mean log2-transformed ratio of the top 15 Hallmark pathways showing the largest changes in Myh10-knockout cells. The bar plot displays the mean log2-transformed ratio (Myh10-knockout/control) for each pathway. Pathways are sorted in descending order by the absolute value of the mean log2-transformed ratio, and the top 15 are presented. Red bars indicate mean log2-transformed ratio > 0 (up-regulation), and blue bars indicate mean log2-transformed ratio < 0 (down-regulation). The number of quantified genes used for calculating the mean in each pathway (*n*) is annotated next to the bar. Red squares mark multiple GO terms pointing to “cell cycle regulation”. (**D**) Heatmap of ssGSEA scores for the top 15 most significantly altered Hallmark pathways in Prrx2-overexpressing cells. The heatmap displays ssGSEA enrichment scores (row Z-score normalized) for each pathway in control and Prrx2-overexpression (Prrx2-OE) samples. Pathways are ordered by ascending padj. Red indicates higher relative enrichment, and blue indicates lower relative enrichment. Red squares mark multiple GO terms pointing to “mitosis and cell cycle”.

## Data Availability

Data available within the article or its Supplementary Materials.

## Supplementary Materials

The following supporting information can be downloaded at: https://www.mdpi.com/article/10.3390/biom16101407/s1, Figure S1: Cluster centroid curves from time-series expression clustering (K-means, k = 7). The x-axis shows time points (0, 7, 14, 28, and 56 days), and the y-axis shows normalized expression values (Z-score). Each colored curve represents the centroid expression trend of one cluster, with different colors indicating the seven distinct temporal expression patterns; Figure S2: Validation of Btg2 mRNA expression changes induced by siRNA knockdown and overexpression. (A,B) RT-qPCR analysis of Btg2 expression after transfection with Btg2 siRNA (A) or overexpression vector (B). Gapdh served as an internal control. **: *p* < 0.01, *n* = 3; Table S1: Statistical tests and exact *p*-values for the comparisons presented in Figure 3, Figure 4 and Figure 5; Table S2: Transcriptome sequencing data (TPM values) from mice exposed to dynamic dust inhalation for 0, 7, 14, 28, and 56 days; Table S3: miRNA sequencing data (counts values) from mice exposed to dynamic dust inhalation for 0, 7, 14, 28, and 56 days; Table S4: Predicted target genes of the screened miRNAs; Table S5: Complete list of miRNA–gene pairs depicted in the Sankey diagrams of Figure 2C,D; Table S6: Transcriptome sequencing data (TPM values) after transfection with miR-146b-5p mimics or Btg2 siRNA in NIH3T3 cells; Table S7: Transcriptome sequencing data (TPM values) after transfection with Btg2 overexpression vector in NIH3T3 cells; Table S8: Quantification of EdU-positive cells across biological replicates; Table S9: Mass spectrometry results of anti-Btg2 immunoprecipitates; Table S10: Mass spectrometry results of anti-Flag immunoprecipitates; File S1: biomolecules-16-01407 original WB figures. The original western blot images can be found in Supplementary Materials. The download link for the Supplementary Tables is: https://doi.org/10.6084/m9.figshare.32894852, accessed on 12 September 2026.
