# Supplementary material for: miR-146b-5p Suppresses Btg2 to Promote Fibroblast Proliferation and Is Associated with Prrx2 and Myh10
Source: Biomolecules. 2026 Sep 28;16(10):1407. doi: 10.3390/biom16101407 (PMC13702142; doi:10.3390/biom16101407)
Supplement: Supplementary file 1 [file biomolecules-16-01407-s001.zip › File S1. biomolecules-4516209 original WB figures.pdf]

Figure4C

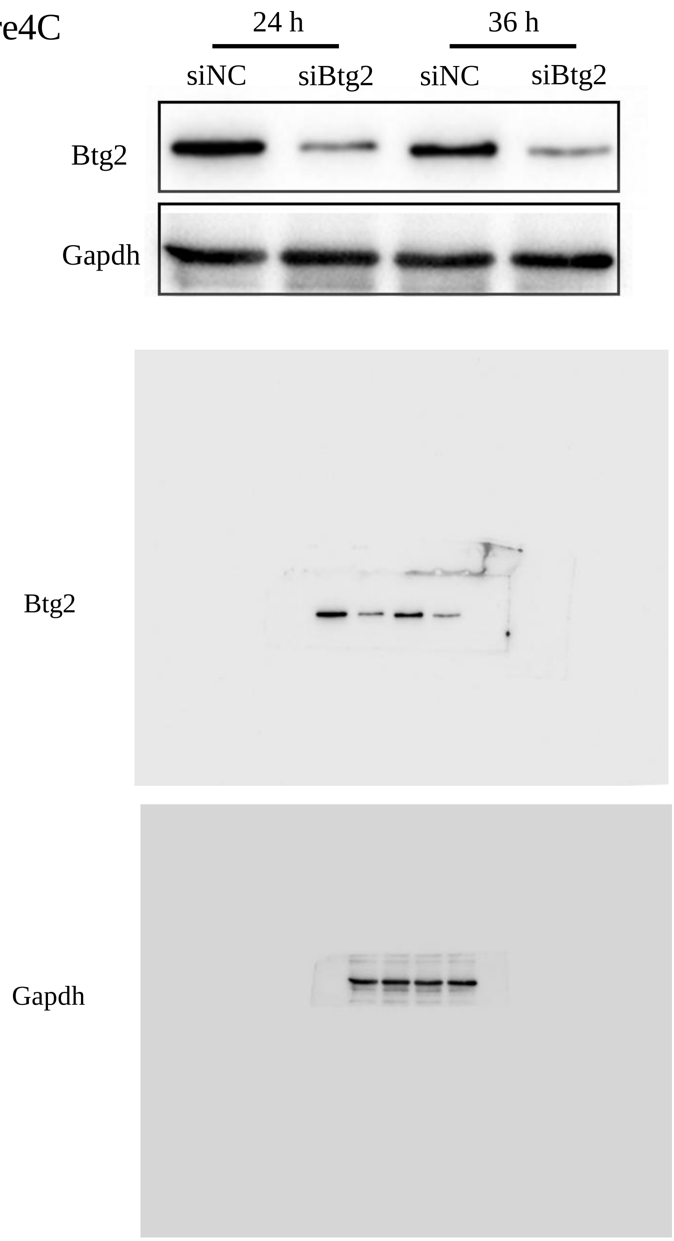

Figure4D

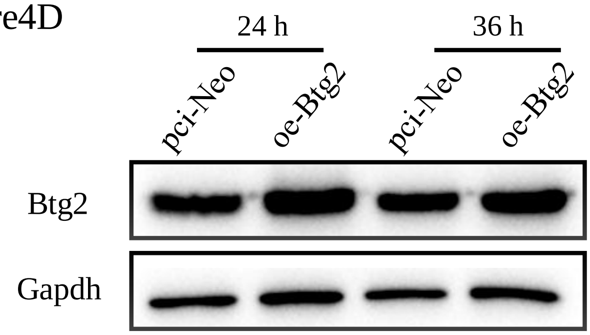

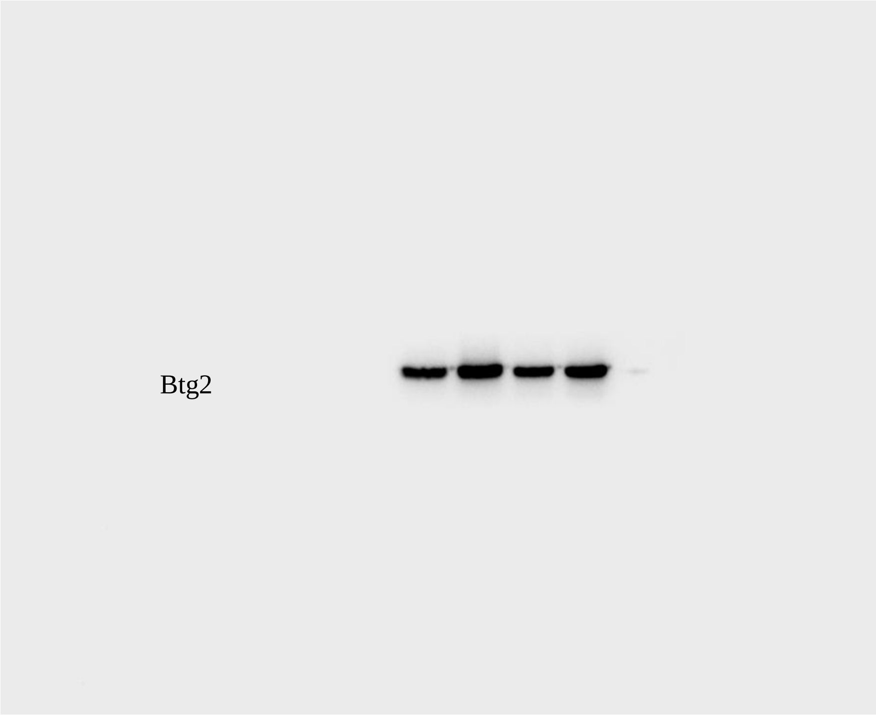

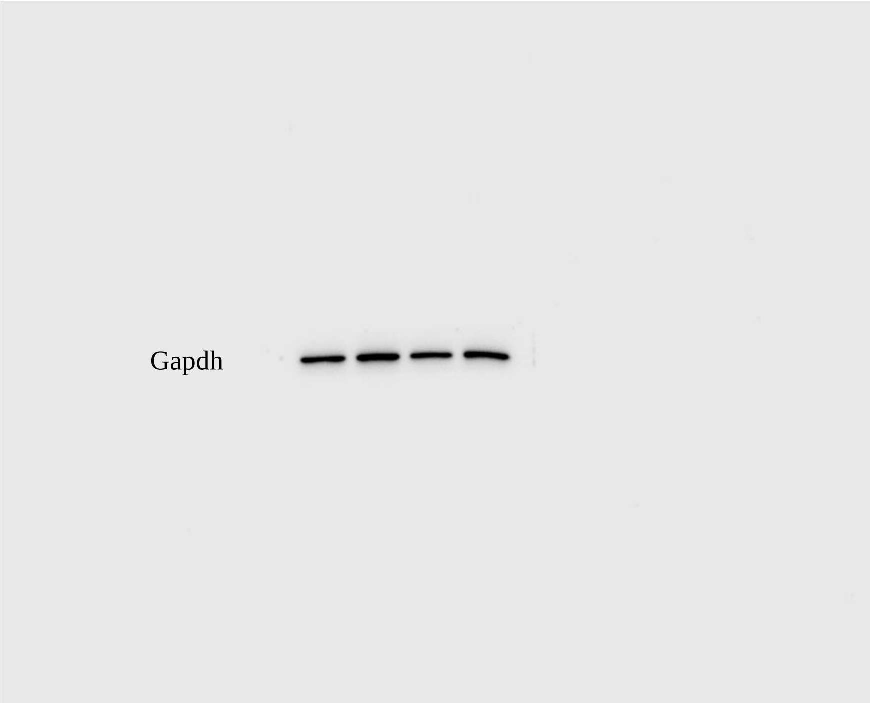

Figure6B

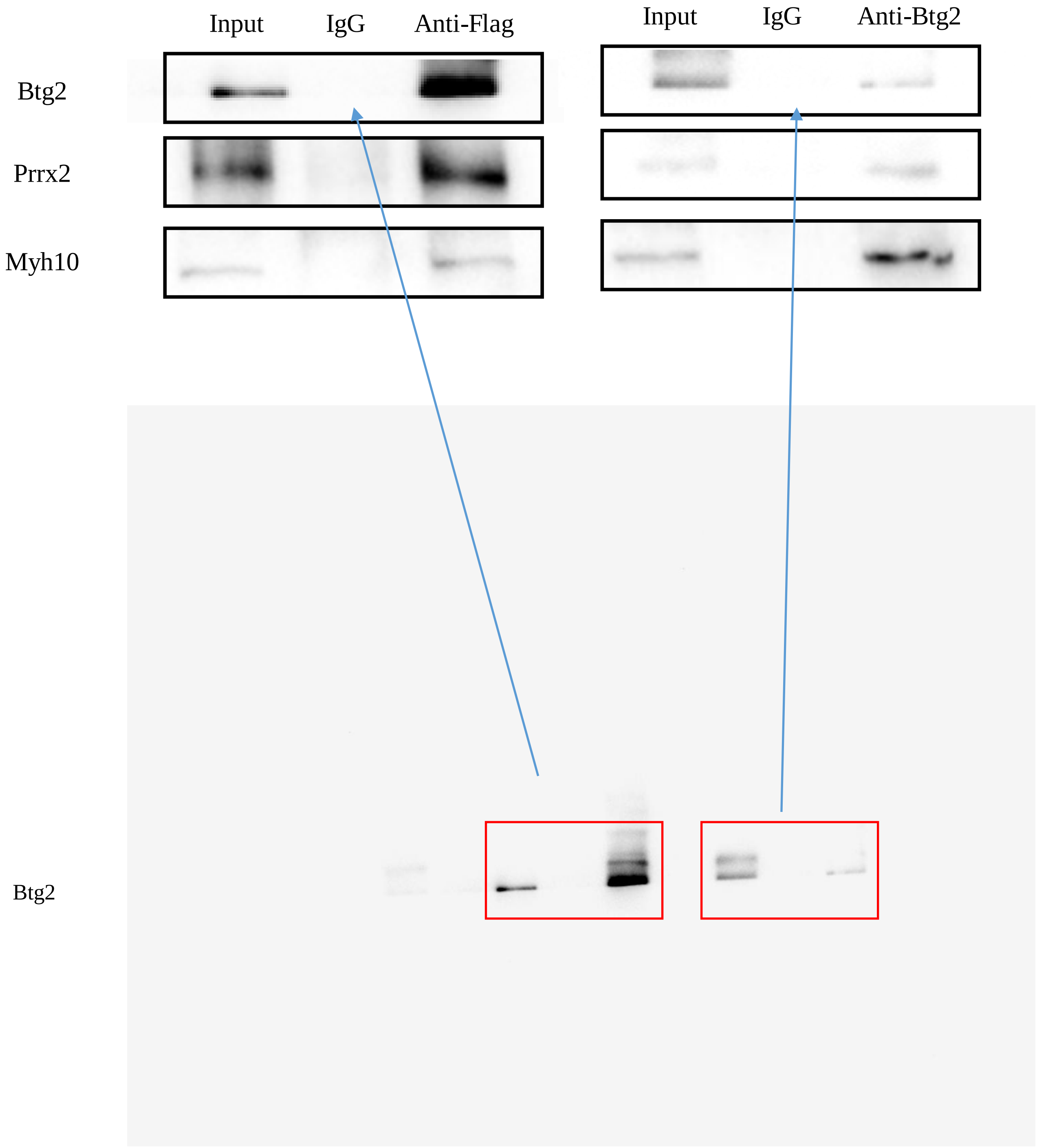
Input
IgG
Anti-Flag
Input
IgG
Anti-Btg2
Btg2
Prrx2
Myh10
Btg2

Figure6B

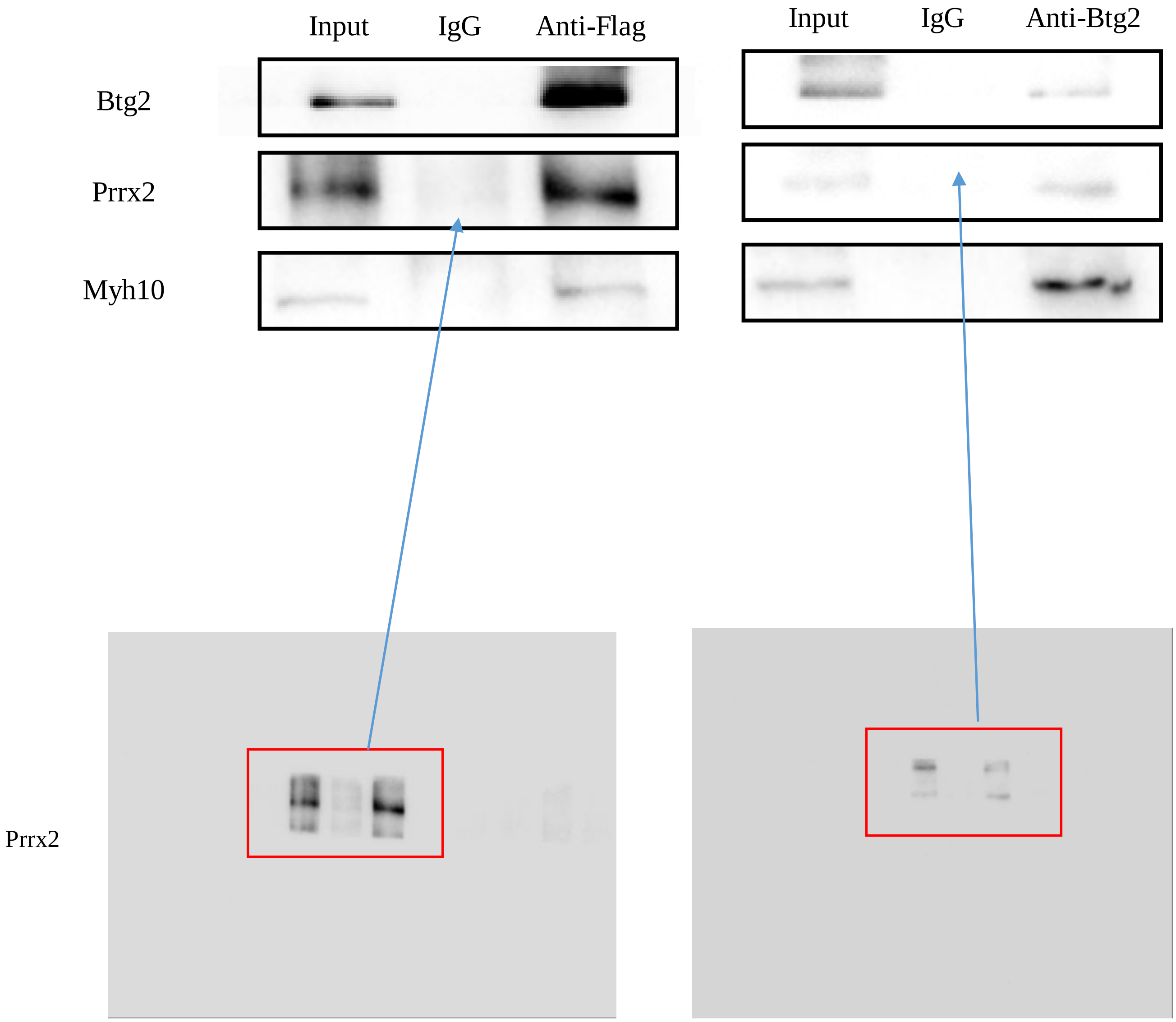
Input
IgG
Anti-Flag
Input
IgG
Anti-Btg2
Btg2
Prrx2
Myh10
Prrx2

Figure6B

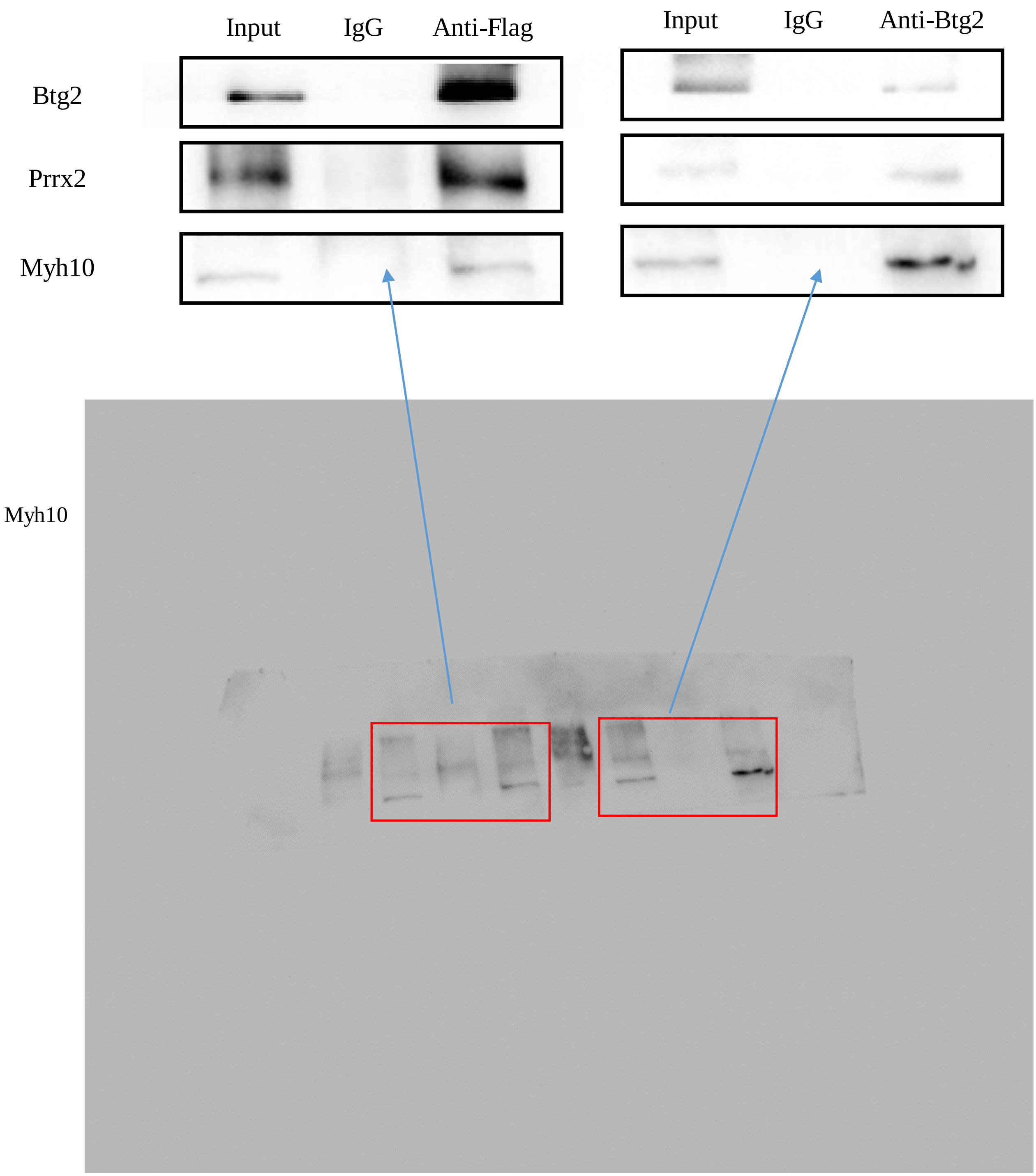
Input
IgG
Anti-Flag
Input
IgG
Anti-Btg2
Btg2
Prrx2
Myh10
Myh10
