# Supplementary material for: miR-146b-5p Suppresses Btg2 to Promote Fibroblast Proliferation and Is Associated with Prrx2 and Myh10
Source: Biomolecules. 2026 Sep 28;16(10):1407. doi: 10.3390/biom16101407 (PMC13702142; doi:10.3390/biom16101407)
Supplement: Supplementary file 1 [file biomolecules-16-01407-s001.zip › Supplementary figures.pdf]

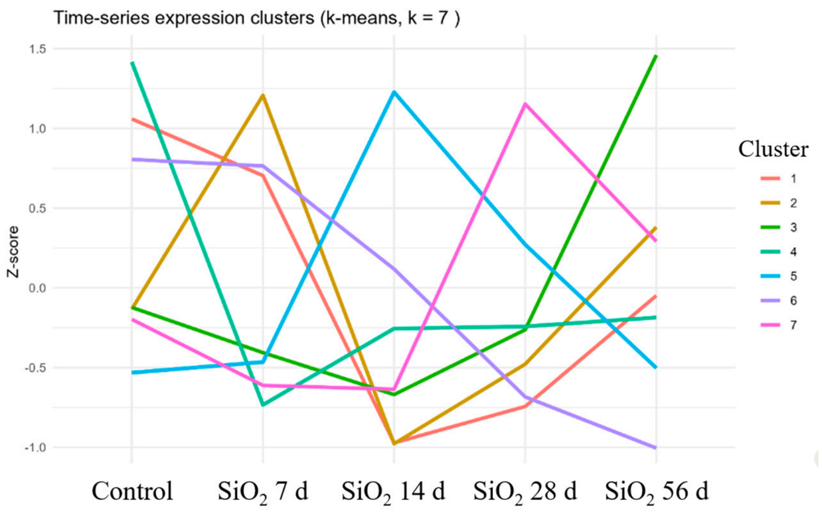

Supplementary Figure S1. Cluster centroid curves from time-series expression clustering (K-means, k = 7). The x-axis shows time points (0, 7, 14, 28, and 56 days), and the y-axis shows normalized expression values (Z-score). Each colored curve represents the centroid expression trend of one cluster, with different colors indicating the seven distinct temporal expression patterns.

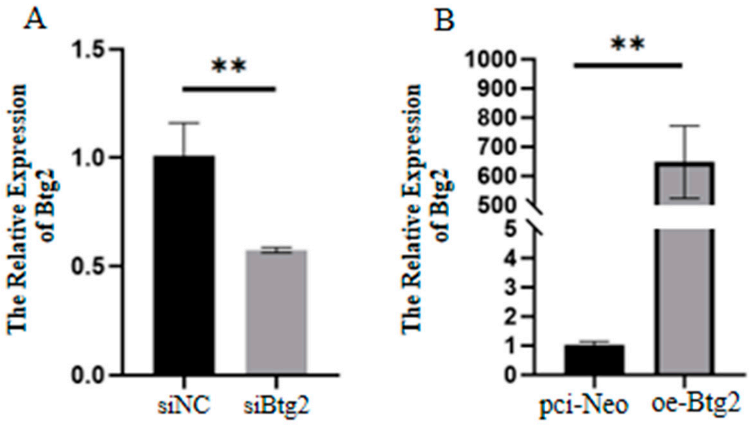

Supplementary Figure S2. Validation of Btg2 mRNA expression changes induced by siRNA knockdown and overexpression. (A–B) RT-qPCR analysis of Btg2 expression after transfection with Btg2 siRNA (A) or overexpression vector (B). Gapdh served as an internal control. **: $p < 0.01$, n=3.
